# Clinical Artificial Intelligence Agents in Nephrology: From Prediction to Action Through Workflow-Native Intelligence—A Roadmap for Workflow-Integrated Care

**DOI:** 10.3390/jcm15072576

**Published:** 2026-03-27

**Authors:** Charat Thongprayoon, Francesco Pesce, Wisit Cheungpasitporn

**Affiliations:** 1 Division of Nephrology and Hypertension, Department of Medicine, Mayo Clinic, Rochester, MN 55905, USA; charat.thongprayoon@gmail.com; 2 Division of Renal Medicine, Ospedale Isola Tiberina-Gemelli Isola, 00168 Rome, Italy; francesco.pesce2@unicatt.it; 3 Department of Translational Medicine and Surgery, Università Cattolica del Sacro Cuore, 00168 Rome, Italy

**Keywords:** artificial intelligence, clinical artificial intelligence agents, nephrology, clinical workflow integration, conceptual roadmap

## Abstract

**Background**: Artificial intelligence in nephrology has largely focused on predictive models for outcomes such as acute kidney injury (AKI), chronic kidney disease (CKD) progression, and transplant complications. Although these models demonstrate technical performance, their real-world clinical impact has remained limited because prediction alone rarely translates into coordinated clinical action. Clinical artificial intelligence agents represent workflow-native systems that operate in real time, interact bidirectionally with clinical environments, adapt to evolving patient and workflow states, and support coordinated clinical action rather than generating isolated predictions. This review proposes clinical artificial intelligence agents as a new paradigm for integrating artificial intelligence directly into nephrology workflows. **Methods**: We conducted a narrative synthesis of emerging literature on artificial intelligence systems, agentic artificial intelligence architectures, clinical decision support, and digital health infrastructures relevant to kidney care. Drawing from interdisciplinary sources in medicine, health informatics, and artificial intelligence research, we developed a conceptual framework describing the architecture, governance requirements, and evaluation principles of clinical artificial intelligence agents in nephrology. **Results**: Clinical artificial intelligence agents represent workflow-integrated systems capable of continuously perceiving patient data, reasoning under clinical constraints, planning tasks, and supporting coordinated clinical actions over time. We describe a layered architecture consisting of perception, cognition, planning and control, action, and learning components. Potential applications span the nephrology care continuum, including CKD management, AKI monitoring, dialysis and continuous renal replacement therapy (CRRT) optimization, kidney transplantation care coordination, glomerulonephritis management, and supervised patient-facing systems. **Conclusions**: Clinical artificial intelligence agents shift the role of artificial intelligence from isolated prediction toward longitudinal clinical orchestration. Future evaluation should prioritize workflow integration, time-to-action, clinician oversight, safety, and patient-centered outcomes rather than relying solely on traditional model performance metrics. This roadmap provides a conceptual foundation for the responsible development and clinical integration of agentic artificial intelligence systems in nephrology.

## 1. Introduction

Chronic kidney disease (CKD) represents a major global public health challenge, affecting an estimated 10–13% of the adult population worldwide and contributing substantially to morbidity, mortality, and healthcare expenditures [1,2]. The progressive nature of CKD, together with its strong association with cardiovascular disease, diabetes, and hypertension, creates complex care needs that require coordinated, multidisciplinary management across the entire disease trajectory [3]. Despite advances in pharmacologic therapies and clinical guidelines, many patients continue to experience delayed diagnosis, suboptimal risk factor control, and fragmented care delivery. These challenges highlight the need for innovative approaches to improve early detection, monitoring, and longitudinal management of kidney disease.

Artificial intelligence (AI) has gained substantial traction in healthcare, including nephrology, with predictive models developed for clinical tasks such as acute kidney injury (AKI) prediction, CKD progression risk stratification, dialysis outcomes assessment, transplant complication monitoring, and diagnostic applications across multiple medical domains [4,5,6,7,8,9,10,11]. These efforts have demonstrated technical feasibility and, in some settings, acceptable predictive performance. However, the clinical impact of model-centric AI has remained limited [5,12,13,14]. A central reason is that prediction alone does not constitute care. Nephrology practice is not driven by isolated risk estimates but by longitudinal interpretation, coordinated action, and continuous adjustment over time, as emphasized in clinical guidelines and studies of CKD care delivery [15,16,17].

AI technologies have increasingly been explored to address these challenges across multiple domains of kidney care [6,18]. In CKD management, machine learning approaches have been applied to identify high-risk patients, predict disease progression, and support clinical decision-making using electronic health record data and real-world datasets [4,8]. Beyond risk prediction, emerging AI-driven systems aim to assist clinicians in optimizing medication management, identifying care gaps, and improving population-level surveillance of kidney disease. AI applications have also been explored in specific domains of kidney care such as nutritional management and glomerular diseases. Nutritional care remains a cornerstone of CKD management, yet providing individualized dietary guidance in routine practice can be challenging. Emerging AI-enabled digital health tools and decision support systems have shown promise in supporting dietary monitoring, personalized nutritional recommendations, and patient engagement in self-management [19]. Similarly, in glomerular diseases, AI techniques have been applied to pathology image analysis, biomarker discovery, and risk stratification, with the goal of improving diagnostic accuracy and supporting precision medicine approaches in complex immune-mediated kidney disorders. Despite these advances, most existing AI applications remain focused on prediction or isolated analytic tasks rather than supporting coordinated clinical workflows.

Recent professional guidance has begun to address the responsible integration of AI into kidney care [4]. A recent statement from the American Society of Nephrology AI Workgroup outlines foundational principles for the development and deployment of AI systems in nephrology, emphasizing patient benefit, transparency, clinician oversight, and careful evaluation of clinical impact. These recommendations highlight the need for AI systems that integrate safely into clinical workflows while preserving human accountability in patient care [4]. In parallel, contemporary clinical guidelines such as the KDIGO 2024 Clinical Practice Guideline for the Evaluation and Management of Chronic Kidney Disease emphasize longitudinal, multidisciplinary care and structured monitoring strategies across the CKD care continuum [15]. These guideline frameworks provide an important clinical context for evaluating how emerging AI systems may support, rather than replace, established models of nephrology care by improving the reliability and timeliness of guideline-concordant interventions.

Nephrology workflows possess several defining characteristics that exceed the functional scope of static predictive models and are well documented in nephrology clinical guidelines and practice literature [5,20,21]. First, kidney care is inherently longitudinal. CKD management unfolds over years and requires repeated reassessment of kidney function trajectories, medication optimization, laboratory surveillance, and timely transitions in care, as emphasized in contemporary CKD clinical guidelines and observational studies of CKD care delivery. Similarly, dialysis management involves ongoing adjustments to dry weight, ultrafiltration, anemia treatment, and access monitoring, often between clinical encounters. Predictive models that generate point-in-time risk scores are poorly suited to these continuous, evolving processes [22,23,24]. Second, clinical decision-making in nephrology is highly contextual and time-sensitive, particularly in AKI [25,26,27]. Decisions regarding fluid resuscitation, nephrotoxin exposure, hemodynamic targets, and consultation timing depend not only on risk estimates but also on dynamic physiologic trends, competing clinical priorities, and care setting. Static alerts or threshold-based decision support systems frequently fail to capture this context, contributing to alert fatigue and limited adoption [28,29,30,31]. Third, kidney care is intrinsically multidisciplinary. Effective management often requires coordination among nephrologists, intensivists, transplant surgeons, pharmacists, nurses, dietitians, and increasingly, patients and caregivers themselves [22,32,33]. Transplant care, in particular, spans pre-transplant evaluation, perioperative management, long-term immunosuppression monitoring, and adherence support, crossing institutional and disciplinary boundaries [34,35,36]. Predictive models, by design, operate in isolation and do not coordinate actions across teams or workflows. Finally, workforce constraints and rising cognitive burden have become defining challenges in nephrology practice [37,38,39]. Expanding patient complexity, increasing administrative demands, and shortages of trained clinicians amplify the need for systems that can actively support clinical work rather than merely inform it [5,38,40]. Tools that require clinicians to interpret outputs, determine next steps, and manually execute actions may paradoxically increase workload instead of alleviating it [41,42]. These limitations reveal a deeper structural mismatch between prevailing AI paradigms and the realities of nephrology practice [5,43]. Model-centric AI approaches, even when technically robust, are designed to generate episodic predictions rather than to participate in care delivery [5]. They do not maintain longitudinal clinical state, coordinate actions across teams, or adapt behavior as care trajectories evolve. As a result, they struggle to meaningfully influence outcomes in a specialty defined by chronic disease management, iterative reassessment, and distributed responsibility [5,43].

Clinical AI agents represent a fundamentally different approach [4,44]. Rather than functioning as isolated analytic tools, clinical AI agents operate as workflow-native systems that continuously perceive clinical data, reason under uncertainty, and support goal-directed action within explicit safety and governance constraints [12,44]. Their clinical value lies not in improved prediction alone, but in their ability to translate recognized risk into timely, coordinated, and accountable care across longitudinal workflows [4,43]. In this context, workflow-native intelligence refers to more than the simple placement of AI tools within existing clinical systems. Rather, it describes intelligent systems designed to function as active components of care delivery by operating in real time, interacting bidirectionally with clinical data and users, adapting to changing patient states and care processes, and helping coordinate actions across longitudinal workflows. This distinguishes workflow-native intelligence from traditional approaches in which AI is merely layered onto clinical practice as an external prediction or recommendation tool that still depends on manual interpretation and separate execution by clinicians. Accordingly, clinical AI agents should not be evaluated or regulated as predictive models. They must instead be assessed and governed as socio-technical systems embedded within real-world nephrology practice, where effectiveness depends on workflow integration, human oversight, safety, equity, and sustained clinical impact [4,45,46].

The primary objective of this review was to examine the emerging concept of clinical AI agents in nephrology and their potential to support workflow-integrated clinical decision-making across the kidney care continuum. The secondary objectives were to outline their core architecture, review potential applications in nephrology, and discuss key considerations related to governance, safety, and evaluation. The central research question was how clinical AI agents can move beyond prediction-based models to support coordinated, workflow-integrated care in nephrology while maintaining appropriate human oversight and safety.

This review examines the architecture, applications, and governance of clinical AI agents in nephrology, positioning agentic systems as a necessary evolution from prediction toward action-oriented clinical intelligence.

## 2. Methods

A narrative review and conceptual synthesis approach was used to examine the emerging literature on AI applications in nephrology and the development of clinical AI agents in healthcare. Relevant publications were identified through a targeted review of the biomedical and health informatics literature, including studies on AI in kidney disease, clinical decision support systems, generative AI, and agentic AI architectures. Sources included peer-reviewed journal articles, clinical guidelines, and interdisciplinary publications addressing AI integration into clinical workflows.

The literature was reviewed and synthesized to develop a conceptual framework describing the architecture, potential clinical applications, governance considerations, and evaluation principles of clinical AI agents in nephrology. Particular attention was given to publications examining AI in CKD, AKI, dialysis care, kidney transplantation, and glomerular diseases, as well as studies discussing human-in-the-loop systems, workflow integration, and safety considerations in clinical AI.

Because the purpose of this manuscript is to provide a conceptual roadmap rather than to conduct a formal systematic review or meta-analysis, the literature selection and synthesis focused on representative developments and key themes relevant to the design, implementation, and evaluation of clinical AI agents in kidney care

## 3. Defining Clinical AI Agents and Agentic AI

**Figure 1:**
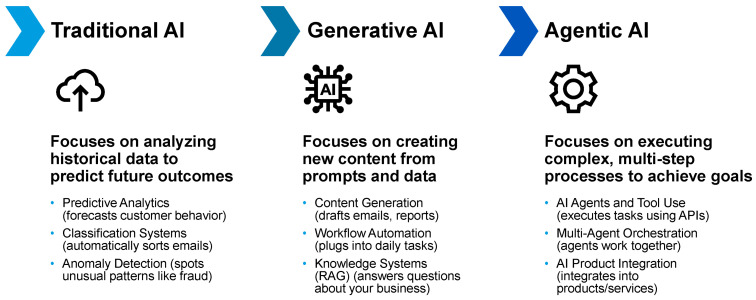
Evolution of artificial intelligence from prediction to agentic clinical systems. Stepwise conceptual diagram illustrating the progression from traditional predictive artificial intelligence to generative artificial intelligence and agentic artificial intelligence. The figure highlights differences in core objective, technical behavior, and clinical role and includes representative nephrology-relevant examples for each stage. AI, artificial intelligence; API, application programming interface; RAG, retrieval-augmented generation.

The term AI agent has been used inconsistently across the biomedical literature, often conflated with predictive models, rule-based clinical decision support systems, or conversational interfaces built on large language models (LLMs) [47,48]. This trajectory from model-centric analytics toward workflow-native, goal-directed systems reflects the broader evolution of AI in healthcare, culminating in agentic AI capable of coordinated clinical action (Figure 1). For clarity, and to enable meaningful evaluation, it is essential to distinguish clinical AI agents from other forms of medical AI based on function rather than underlying algorithms.

Agentic AI represents a broader computational paradigm in which software systems pursue defined goals through continuous perception, reasoning, planning, and action within dynamic environments. In healthcare, this paradigm can be implemented through clinical AI agents, which represent a domain-specific instantiation of agentic AI designed to operate within clinical workflows and healthcare information systems. While agentic AI describes the general architecture of goal-directed intelligent systems, clinical AI agents apply these principles to patient care by integrating longitudinal clinical data, medical knowledge, and explicit safety constraints to support coordinated clinical decision-making over time. Importantly, clinical AI agents should be distinguished from related technologies that are often conflated with them. Medical robots primarily refer to physical systems that assist with procedural or mechanical tasks, whereas traditional clinical decision support systems typically provide rule-based alerts or recommendations without persistent context or goal-directed planning. In contrast, clinical AI agents function as workflow-native software systems capable of maintaining longitudinal clinical context, coordinating tasks, and supporting human clinicians within predefined governance and safety frameworks.

### 3.1. What Is a Clinical AI Agent?

A clinical AI agent is a software system designed to operate within healthcare environments that continuously pursues clinical objectives by integrating perception, reasoning, planning, and action under explicit constraints [49,50,51]. Unlike static models that generate isolated predictions, a clinical AI agent functions as an ongoing process embedded in clinical workflows [49,50,51].

Specifically, a clinical AI agent:

Continuously perceives clinical data

The agent maintains persistent awareness of relevant patient information, including structured data (laboratory values, medications, vital signs), unstructured data (clinical notes, reports), device-generated data (dialysis machines, monitors), and, where applicable, patient-reported or remotely collected inputs [12,52,53,54]. This continuous perception enables longitudinal state tracking rather than episodic inference.

Reasons using medical knowledge and uncertainty

The agent integrates domain-specific medical knowledge, clinical guidelines, and probabilistic reasoning to interpret observed data [44,55]. Rather than producing a single risk estimate, it evaluates evolving clinical states, incorporates uncertainty, and contextualizes recommendations within patient-specific factors and care settings [44,56].

Plans and executes actions

Based on its internal assessment and predefined objectives, the agent can decompose clinical goals into actionable steps, prioritize tasks, and execute or propose actions within permitted scopes [44,46,57,58]. These actions may include generating clinical documentation, triaging messages, prompting guideline-concordant interventions, coordinating referrals, or escalating cases to human clinicians [59]. Crucially, execution occurs within predefined safety boundaries and institutional policies [59].

Learns from feedback and outcomes

Clinical AI agents incorporate feedback from clinician interactions, overrides, and patient outcomes to refine future behavior [55,60]. Learning may occur through structured performance evaluation, reinforcement mechanisms, or supervised updates, allowing the system to adapt to local practice patterns while remaining aligned with clinical standards [55].

Operates under explicit goals and constraints

The agent’s behavior is guided by clearly defined clinical objectives, such as slowing CKD progression, preventing avoidable AKI, or optimizing dialysis safety [4,61,62]. Equally important are constraints that limit autonomy, enforce human oversight, and ensure regulatory compliance [4,61,62]. These constraints distinguish clinical agents from unrestricted autonomous systems. This combination defines clinical AI agents as workflow-native, goal-directed systems rather than analytic tools or passive decision aids [61,62]. The distinction is particularly relevant in nephrology, where effective care depends on sustained monitoring, coordinated actions across teams, and continuous adaptation to changing clinical trajectories.

### 3.2. Agentic AI Versus Existing Clinical AI Paradigms

The rapid expansion of AI in healthcare has led to conceptual ambiguity, with predictive models, clinical decision support systems (CDSS), LLM–based chatbots, and agentic systems often discussed interchangeably [48,51,63]. Clarifying these distinctions is essential, as differences in functionality, autonomy, and workflow integration have direct implications for safety, evaluation, and regulation [4,64]. In nephrology, where care is longitudinal and action-oriented, these distinctions are particularly consequential [4].

Table 1 contrasts dominant AI paradigms currently encountered in nephrology practice. Predictive machine learning (ML) models generate probabilistic estimates at discrete time points but do not maintain state, initiate actions, or adapt workflows. Traditional CDSS rely on predefined rules and thresholds, offering limited contextual awareness and minimal adaptability. LLM-based chatbots can generate fluent clinical text and respond interactively but lack persistent goals, authority to act, and formal learning from clinical outcomes.

By contrast, clinical AI agents are defined by temporal continuity, goal-directed behavior, and the ability to coordinate actions within workflows under explicit constraints [49,65]. While autonomy may vary by implementation, agentic systems are uniquely capable of maintaining longitudinal context, decomposing clinical objectives into tasks, and adapting behavior based on feedback [49]. These properties distinguish agentic AI from prior generations of clinical AI and justify its consideration as a separate class of clinical systems rather than an incremental extension of existing tools [50,65].

## 4. Architecture of Clinical AI Agents in Nephrology

Clinical AI agents are best understood as layered systems that transform heterogeneous clinical data into coordinated, goal-directed actions under explicit constraints [49,65]. This architecture distinguishes agentic systems from traditional analytic tools by enabling persistent context, task orchestration, and adaptive behavior over time [50,65]. In nephrology, where care is longitudinal, data rich, and workflow intensive, each architectural layer plays a distinct and clinically meaningful role [4].

### 4.1. Perception Layer

**Figure 2:**
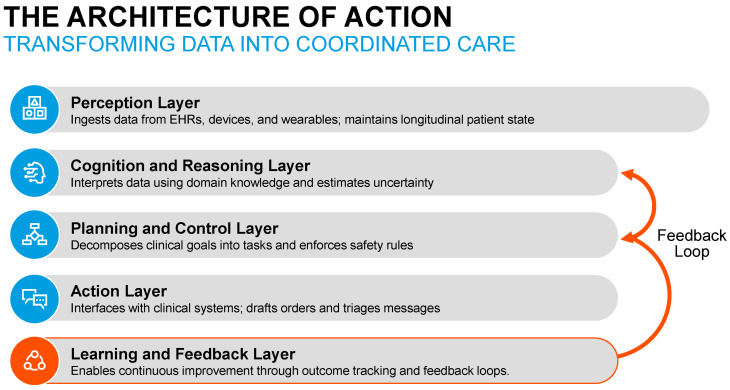
Architecture of action in clinical AI agents. Layered closed-loop schematic illustrating how clinical AI agents transform longitudinal nephrology data into coordinated, goal-directed actions through perception, reasoning, planning, action, and learning. Representative nephrology-specific inputs, outputs, and inter-layer interactions are shown to emphasize workflow-native operation.

The perception layer provides continuous situational awareness by ingesting and updating patient state from multiple data streams (Figure 2) [50,54,65]. Core inputs include structured electronic health record data (laboratory results, medications, vital signs), unstructured clinical text (progress notes, consults, pathology reports), and device-generated telemetry from dialysis machines and monitoring systems [54]. Increasingly, this layer may also incorporate data from wearables and remote patient monitoring platforms, such as blood pressure trends, weight trajectories, or patient-reported symptoms [50,65].

Unlike episodic data pulls used by predictive models, the perception layer maintains persistent, longitudinal state, allowing the agent to track trends, detect deviations, and contextualize changes across time and care settings [50,65].

### 4.2. Cognition and Reasoning Layer

The cognition layer interprets perceived data using a combination of statistical inference, symbolic reasoning, and domain knowledge [49]. Foundation models, whether text based or multimodal, enable synthesis of heterogeneous inputs, while nephrology specific knowledge representations encode clinical concepts, relationships, and constraints [50,65]. Importantly, reasoning is guided by clinical guidelines and institutional policies, ensuring that outputs remain aligned with accepted standards of care [50,65].

Uncertainty estimation is a critical component of this layer. Rather than producing deterministic recommendations, the agent assesses confidence, identifies ambiguity, and modulates downstream actions accordingly, such as deferring to human judgment when uncertainty is high [50,54,65].

### 4.3. Planning and Control

The planning and control layer translates clinical goals into executable workflows [50,65]. This includes decomposing high-level objectives, such as preventing intradialytic hypotension or optimizing chronic kidney disease management, into discrete tasks; prioritizing actions under competing constraints; and sequencing steps over time [4]. Safety rules and guardrails are enforced at this stage, limiting permissible actions and ensuring compliance with regulatory and institutional requirements.

Escalation logic is central to safe deployment. The agent determines when to proceed autonomously within its allowed scope and when to defer, notify, or transfer control to clinicians, preserving human oversight and accountability [50,65].

### 4.4. Action Layer

The action layer interfaces directly with clinical systems and users [50,54,65]. Actions may include drafting orders or documentation for clinician review, triaging inbox messages, coordinating referrals or follow-up testing, and communicating with patients through approved channels. In all cases, actions are executed within predefined authorization boundaries, with transparency and traceability [66].

Crucially, this layer enables workflow integration, allowing the agent to reduce administrative burden and close the loop between assessment and intervention rather than merely generating recommendations [50,54,65].

### 4.5. Learning and Feedback

The learning and feedback layer enables continuous improvement while maintaining safety [50,54,65]. Agent performance is evaluated through outcome tracking and process metrics, such as timeliness of interventions or rates of clinician override. Drift detection mechanisms monitor changes in data distributions, practice patterns, or system behavior that may degrade performance over time.

Adaptive learning in clinical AI agents must occur within clearly defined governance and safety boundaries [4,6]. Although the ability to learn from feedback and outcomes is often cited as a defining property of agentic systems, unrestricted adaptation during routine clinical operation may introduce unacceptable risks in healthcare environments. In practice, most clinical implementations are expected to rely on controlled learning processes in which model updates occur through supervised evaluation cycles rather than continuous unsupervised online learning.

Several modes of learning may be relevant in this context [4,6]. Offline learning refers to periodic retraining or recalibration using curated datasets followed by retrospective evaluation before deployment of an updated model. Supervised adaptive learning incorporates structured feedback signals such as clinician overrides, confirmed outcomes, or systematic error analyses to guide targeted system refinement [4,6]. By contrast, unrestricted online learning that modifies system behavior in real time during clinical deployment is unlikely to be appropriate for high-risk medical decision environments unless strict governance safeguards are in place.

To mitigate unintended behavioral drift, clinical AI agents should operate within a framework of continuous monitoring and controlled updating [4,6]. Drift detection mechanisms can track changes in input data distributions, evolving clinical practice patterns, or shifts in model outputs that may degrade performance over time. When such drift is detected, institutions may initiate formal review processes that include performance re-evaluation, recalibration, or supervised retraining before redeployment.

Importantly, adaptation in clinical AI agents should remain transparent and auditable. Decision logs, performance dashboards, and structured monitoring of clinician overrides can provide signals that identify emerging system weaknesses or unintended behavior. Within this framework, learning becomes a governed lifecycle process rather than an autonomous system property, ensuring that adaptive improvement occurs without compromising safety, reliability, or clinician oversight [4,6].

These layers form a closed-loop clinical system that senses, reasons, acts, and adapts over time [50,54,65]. This architecture underpins the ability of clinical AI agents to function as workflow-native intelligence in nephrology [4,6], supporting sustained care delivery rather than isolated decision points. Within this governance framework, adaptive learning becomes a controlled lifecycle process rather than an unrestricted system capability, ensuring that performance improvements occur through supervised evaluation and institutional oversight rather than unsupervised real-time modification during clinical operation.

## 5. Applications Across the Nephrology Care Continuum

### 5.1. CKD

CKD represents a setting in which the primary challenge is not diagnostic uncertainty but reliable execution of evidence-based care over extended time horizons [8,67]. While predictive models can estimate progression risk, they do not ensure that guideline-concordant interventions are initiated, monitored, and sustained as patient status evolves [68].

Recent studies have explored AI-enabled longitudinal monitoring systems for CKD populations using electronic health record data [67,69,70,71]. For example, ML-based risk monitoring platforms applied to large health system datasets, often involving tens of thousands of patients, have been used to identify individuals at high risk for rapid CKD progression and to prompt earlier nephrology referral or medication optimization [67,69,70,71]. In these studies, key outcome indicators included earlier identification of high-risk patients and reductions in time to clinical intervention following risk detection.

**Figure 3:**
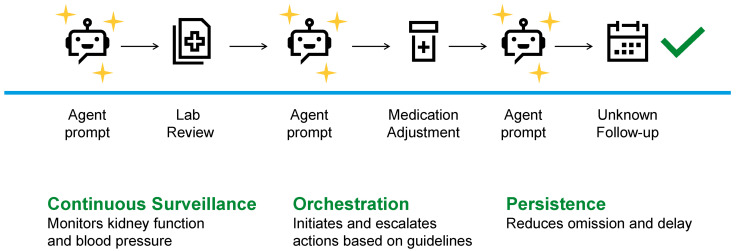
Longitudinal care orchestration by a clinical AI agent in chronic kidney disease. Process-oriented diagram showing how a clinical AI agent may support surveillance, identification of care gaps, guideline-based prompting, and follow-up over time, with explicit clinician interaction points.

Clinical AI agents address this gap by operationalizing clinical guidelines into executable longitudinal workflows [72]. Agents track kidney function trajectories, albuminuria status, blood pressure control, medication exposure, and laboratory surveillance requirements (Figure 3), identifying when patients meet eligibility criteria for specific interventions and when reassessment is warranted [72,73]. Importantly, agents support persistence: ensuring that therapies are not only started, but revisited, adjusted, or discontinued as clinical circumstances change.

In CKD care, the defining contribution of clinical AI agents is operational reliability [4,5]. Their value lies in reducing omission, delay, and fragmentation in long-term disease management rather than refining risk stratification alone.

### 5.2. AKI

AKI is characterized by rapid physiologic change and narrow windows for intervention [6,18]. Traditional alert-based systems often fail in this context by generating high volumes of non-specific notifications that compete for clinician attention without clear prioritization [4].

Several prospective and retrospective studies have evaluated AI-based early warning systems for AKI in hospitalized patients [13,74,75,76,77]. In large cohort analyses involving thousands of admissions, machine learning models integrated with electronic health record systems have demonstrated the ability to detect AKI risk hours to days before conventional clinical recognition [13,74,75,76,77]. Implementation studies have reported reductions in time to clinical evaluation and improvements in guideline-concordant management following automated alerts.

**Figure 4:**
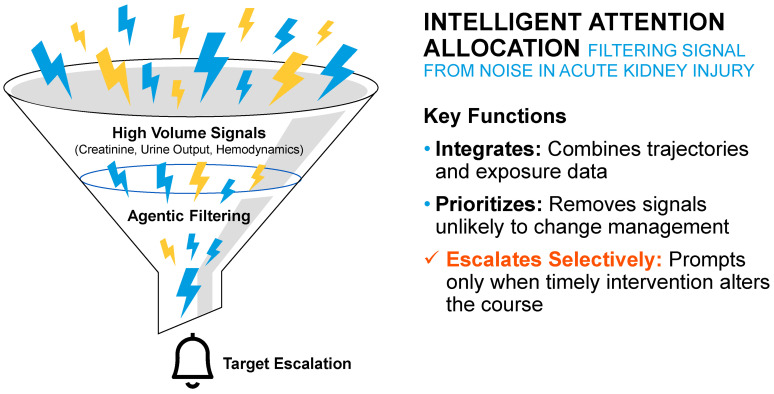
Intelligent attention allocation in acute kidney injury. Workflow diagram illustrating how a clinical AI agent may continuously integrate physiologic and laboratory signals, prioritize actionable deterioration patterns, and selectively escalate cases to clinicians in time-sensitive care settings.

Clinical AI agents support AKI management through time-critical selective escalation (Figure 4). By continuously integrating creatinine trajectories, urine output trends, hemodynamics, medication exposure, and baseline vulnerability, agents identify situations in which timely intervention is most likely to alter clinical course [8,18]. Signals unlikely to change management are deprioritized, while patterns suggestive of progressive or modifiable injury prompt targeted escalation.

The distinguishing capability in AKI is not prediction of risk, but intelligent attention allocation. Agents help ensure that clinical focus is directed toward patients and moments where action is most consequential [18,78].

### 5.3. Dialysis and Continuous Renal Replacement Therapy

Dialysis and continuous renal replacement therapy (CRRT) generate clinically meaningful signals across repeated treatments rather than within isolated encounters [18,79]. Although protocols exist for common complications, recognition of evolving patterns often depends on manual synthesis that is difficult to sustain in high-volume environments.

AI approaches have also been explored in dialysis and critical care environments [80,81]. For instance, predictive models embedded within dialysis monitoring systems have been evaluated for the early detection of intradialytic complications or fluid imbalance, while machine learning-assisted CRRT monitoring systems have been studied for optimizing therapy parameters and improving treatment stability in critically ill patients [79,80,81,82]. These studies typically involve retrospective cohort designs using large dialysis or intensive care databases and report outcomes such as improved prediction accuracy, earlier detection of complications, or enhanced treatment monitoring.

Clinical AI agents contribute by performing between-session synthesis. Agents aggregate intradialytic hypotension events, ultrafiltration tolerance, blood pressure trends, laboratory trajectories, and treatment interruptions to identify patterns that warrant reassessment [18,79,83]. This supports incremental refinement of dry weight, ultrafiltration strategy, and anemia management while remaining within established clinical frameworks.

In CRRT, agents can assist with early recognition of inadequate clearance, filter dysfunction, or emerging hemodynamic intolerance, prompting review without replacing clinician judgment [5,12,18]. Here, the agent’s value lies in maintaining continuity of insight across treatments rather than automating individual decisions.

### 5.4. Kidney Transplantation

Kidney transplantation spans multiple phases of care and involves coordination among transplant surgeons, nephrologists, pharmacists, nurses, and patients across institutional boundaries [4,84,85]. No single clinician maintains complete situational awareness, and failures in coordination can have disproportionate clinical consequences. Clinical AI agents support transplantation by maintaining cross-team continuity. Agents track immunosuppression regimens, drug levels, kidney function trends, infection surveillance, and protocol milestones, prompting reassessment when deviations occur or follow-up steps are missed [4,85]. They can also assist with logistical alignment, such as synchronizing laboratory monitoring with clinic visits or identifying gaps in surveillance.

The primary contribution of agents in transplantation is not decision replacement [4,85], but improved reliability across distributed workflows, ensuring that critical actions occur consistently despite fragmentation of care.

### 5.5. Glomerulonephritis

Glomerulonephritis management is defined by diagnostic heterogeneity, limited evidence, and evolving disease behavior [86,87]. Clinical decisions frequently depend on recognizing when accumulating data warrant reconsideration of diagnosis or therapy rather than continuation of the current course.

Clinical AI agents support glomerulonephritis care by enabling formal reassessment under uncertainty. Agents track disease activity markers, immunosuppressive exposure, biopsy findings, and response trajectories, prompting structured re-evaluation when observed patterns diverge from expected courses [4,86,87]. This ensures that uncertainty is revisited deliberately rather than implicitly tolerated.

In this domain, agents do not resolve ambiguity. They provide a framework for consistent, transparent reassessment in conditions where clinical judgment remains central.

### 5.6. Patient-Facing Agents

Patient-facing clinical AI agents function as supervised extensions of clinician workflows rather than independent advisors [48,88]. Their defining capability is protocol-governed engagement aligned with clinician-defined goals.

Agents can support symptom triage, home data review, and adherence monitoring within predefined parameters, escalating concerns to clinicians when thresholds are met [88,89]. By embedding patient interactions within the same governance, auditability, and escalation framework as clinician-facing agents, these systems enhance continuity without introducing unsupervised autonomy [89,90].

## 6. Technical and Infrastructural Barriers to Implementation

While the architectural framework for clinical AI agents is well-defined, realized implementation in nephrology faces significant technical hurdles [4]. Unlike predictive models that can function on retrospective data extracts, agentic systems require real-time, bi-directional integration with the electronic health record (EHR) and a degree of reliability that probabilistic models struggle to guarantee. The transition from “human-in-the-loop” to “human-on-the-loop” cannot occur without resolving these foundational challenges [4].

### 6.1. The Challenge of Deep Integration: Interoperability and Write-Back

The primary barrier to deploying workflow-native agents is the “last mile” of integration. To function safely and effectively, a clinical AI agent must not only read clinical data but also interact with healthcare information systems in a controlled and auditable manner. In practice, this requires bidirectional interoperability between the agent and the electronic health record, often referred to as read-write or “write-back” capability [4].

Interoperability standards are central to this architecture. Standards such as Fast Healthcare Interoperability Resources (FHIR) allow structured clinical data, including laboratory results, medications, and encounter information, to be accessed and exchanged across systems using standardized formats [4,19]. Through secure FHIR-based application programming interfaces, clinical AI agents can retrieve relevant patient data and generate structured outputs that are inserted into clinical workflows in a transparent and auditable manner. In many implementations, write-back functions remain limited to preparatory actions, such as generating draft documentation or suggested orders, while final authorization remains under clinician control.

Early real-world implementations of AI-enabled clinical systems have begun to adopt similar architectural patterns, particularly in population health management platforms and EHR integrated decision support systems [4,19]. These implementations demonstrate that workflow-integrated AI requires not only algorithm development but also robust interoperability infrastructure, governance controls, and carefully designed integration layers that allow intelligent systems to operate safely within complex healthcare information environments.

### 6.2. Reliability at Scale: Context Windows and Probabilistic Reasoning

Technical constraints within the underlying foundation models also pose risks to clinical reliability. Similarly, technical limitations of LLM–based systems may affect reliability in longitudinal nephrology care. Decisions regarding immunosuppression management, vascular access planning, or recurrent AKI often depend on clinical events distributed across multiple encounters over time [4,19]. When relevant historical context is not retrieved or exceeds the effective context window of the model, the system may generate incomplete reasoning, overlook important contraindications, or produce recommendations that require clinician correction. These examples underscore the need for robust retrieval pipelines, deterministic safeguards, and human oversight in the implementation of clinical AI agents [19]. Nephrology relies heavily on longitudinal history; a decision regarding immunosuppression or vascular access often depends on events that occurred months prior [4]. Current LLMs face limitations in “context window”—the amount of information they can process simultaneously. To mitigate this, agents must employ sophisticated Retrieval-Augmented Generation (RAG) strategies that accurately surface relevant history without hallucinating connections that do not exist [6,18].

Furthermore, the probabilistic nature of generative AI introduces the risk of logic inaccuracy [4]. Unlike rule-based CDSS, which are deterministic, an agent might correctly identify a hyperkalemia event but generate unsupported or clinically incorrect recommendations for management or fail to recognize a contraindication due to probabilistic noise. Ensuring safety requires hybrid neuro-symbolic architectures, where the flexible reasoning of the agent is bounded by deterministic code (e.g., hard-coded safety checks that strictly forbid potassium prescription if levels exceed 5.5 mEq/L) that cannot be overridden by the model’s generative logic.

## 7. Governance, Safety, and Regulation

The introduction of clinical AI agents into nephrology raises governance considerations that extend beyond those encountered with predictive models or traditional clinical decision support systems [68,91,92,93,94,95]. Because agentic systems can perceive, reason, and act over time within clinical workflows, their safety and effectiveness depend not only on algorithmic performance but also on how they interact with clinicians, patients, and institutional processes [68,91,92,93,94,95]. Robust governance frameworks are therefore essential for responsible deployment.

### 7.1. Human-in-the-Loop Design

Human oversight is a foundational requirement for clinical AI agents [90]. Agent behavior must be bounded by explicit approval thresholds that define which actions may be executed automatically, which require clinician confirmation, and which mandate escalation [46]. These thresholds should be task-specific and risk-adjusted, reflecting the clinical consequences of potential errors [96].

In clinical practice, the scope of permissible actions for clinical AI agents should be stratified according to clinical risk and potential patient impact [4,18]. Low-risk operational tasks may be executed automatically within predefined institutional guardrails, such as monitoring laboratory values and physiologic trends, identifying missing tests, organizing longitudinal clinical data, preparing draft documentation, or generating workflow prompts. These actions support information management and care coordination without directly altering patient treatment [4,18]. Tasks with moderate clinical implications should require clinician confirmation before execution. Examples include proposing medication adjustments based on guideline criteria, recommending nephrology consultation for declining kidney function, or suggesting additional diagnostic testing. In these situations, the clinical AI agent generates structured recommendations or workflow actions that must be reviewed and approved by a clinician.

High-risk clinical decisions that directly affect diagnosis or treatment should remain under clinician authority [4,18]. Examples include initiating or discontinuing immunosuppressive therapy, modifying dialysis prescriptions with hemodynamic implications, or escalating care for critically ill patients. In these contexts, clinical AI agents function strictly as decision-support systems rather than executing actions independently. Importantly, these authorization thresholds may vary by clinical context, institutional governance, and system maturity [4,18]. For example, inpatient critical care settings may require stricter approval thresholds than population-level chronic disease monitoring. Clear escalation rules, auditability, and clinician override mechanisms are therefore essential to ensure safe deployment while preserving clinician accountability.

Override mechanisms are equally critical. Clinicians must be able to interrupt, modify, or reject agent-generated actions at any point, with minimal friction [90]. Overrides should not be treated as system failures but as expected components of safe human–machine collaboration. Furthermore, safety requires hybrid architectures. While the agent’s reasoning may use probabilistic LLMs, critical constraints (e.g., ‘do not order nephrotoxins if eGFR < 30’) must be hard-coded as deterministic guardrails that cannot be overridden by the model’s generative logic.

Clear responsibility attribution is necessary to preserve clinical accountability. Governance frameworks must specify how responsibility is shared among clinicians, institutions, and system developers, particularly when agents operate across multiple steps of a workflow [46,90]. Explicit attribution supports trust, medicolegal clarity, and ethical deployment.

### 7.2. Auditability and Transparency

Agentic systems require a higher standard of transparency than static models [46]. Decision logs should record the inputs, reasoning pathways, and constraints that led to each recommendation or action [97]. These logs enable retrospective analysis and support quality assurance, research, and regulatory review.

Action traceability ensures that all agent-initiated or agent-supported actions can be linked to specific system states and decision points. This traceability is essential in complex workflows, such as dialysis management or transplant care, where outcomes emerge over time and across teams [4,97,98].

Post hoc review mechanisms allow institutions to examine agent performance after clinical events, near misses, or unexpected outcomes [46,97]. Such reviews should focus not only on algorithmic reasoning but also on interaction with clinicians, timing of actions, and appropriateness of escalation.

### 7.3. Regulatory Considerations

Current regulatory frameworks for AI in healthcare have largely evolved around model-centric paradigms, emphasizing algorithm validation, performance metrics, and static risk classification [46]. While appropriate for predictive models, these approaches are insufficient for agentic systems that operate as continuous, adaptive processes within clinical environments.

Clinical AI agents require process-level evaluation, encompassing how data are perceived, how decisions unfold over time, how actions are constrained, and how humans interact with the system. Safety and effectiveness cannot be assessed solely through accuracy or discrimination metrics but must include workflow impact, failure modes, and governance controls [99].

Alignment with evolving software as a medical device (SaMD) frameworks will be necessary [99,100], but additional guidance is likely required to address autonomy, learning over time, and multi-agent coordination. Nephrology, with its longitudinal care models and high reliance on protocols, provides an instructive setting for developing and testing such regulatory approaches [4].

Clinical AI agents must be evaluated and governed as socio-technical workflows, not isolated algorithms [101]. Their safety and value emerge from the interaction between technology, clinicians, patients, and organizational structures. Recognizing this distinction is essential for responsible integration of agentic AI into nephrology practice.

## 8. Research Agenda and Evaluation Metrics

The evaluation of clinical AI agents requires a fundamental departure from model-centric assessment paradigms [102,103]. Metrics traditionally used to evaluate predictive models, such as discrimination or calibration, are necessary but insufficient for agentic systems that operate continuously, interact with clinicians, and influence care delivery over time [103]. Clinical AI agents should therefore be evaluated as workflow-integrated socio-technical systems, not as standalone algorithms. At a minimum, evaluation should include a standardized set of core domains reflecting real-world system behavior within clinical workflows: process performance, safety, human oversight, equity, and sustainability.

### 8.1. Why Area Under the Receiver Operating Characteristic Curve (AUROC) Should Not Be the Primary Endpoint for Clinical AI Agents

Area under the receiver operating characteristic curve has become the dominant performance metric in clinical AI research [103,104]. While AUROC is appropriate for evaluating discrimination in static classification tasks, it is fundamentally misaligned with the purpose and behavior of clinical AI agents [103,104]. Agentic systems are not designed to optimize classification accuracy at a single time point; they are designed to translate evolving clinical signals into timely, appropriate action within real-world workflows [103,104].

An agent may exhibit excellent discrimination yet fail clinically if its outputs arrive too late, are poorly prioritized, or do not trigger meaningful downstream action [45]. This reflects the ‘last mile’ problem in clinical AI: a high-accuracy prediction is useless if it cannot bridge the gap to execution. AUROC measures the potential for information, whereas agentic evaluation must measure the successful completion of a workflow. Conversely, an agent with modest predictive performance may produce substantial clinical benefit if it reliably accelerates appropriate intervention, reduces omissions, or improves coordination of care. AUROC is agnostic to these distinctions [46].

For these reasons, we argue that AUROC should not serve as the primary endpoint for evaluating clinical AI agents [103,104]. At most, discrimination metrics should function as supporting evidence that an agent’s internal risk assessments are technically sound [103,104]. They do not, on their own, demonstrate clinical value.

### 8.2. Process-Level Endpoints as Co-Primary Outcomes

Evaluation of clinical AI agents should prioritize process-level metrics that directly reflect system behavior within clinical workflows [104]. We propose that process metrics serve as co-primary endpoints in agentic AI research. At a minimum, evaluation should include process performance, safety, human oversight, equity, and sustainability as a shared operational framework. Process performance should assess whether the system improves the timeliness and completion of clinically meaningful actions, such as time-to-action, response latency, and completion of intended workflow steps. Safety should include near-miss events, failure-to-escalate events, inappropriate recommendations or actions, and breakdowns in human–machine handoff. Human oversight should be assessed through clinician override frequency, override context, and measures of trust calibration, since appropriate human control remains central to safe deployment. Equity should examine whether performance, escalation, or follow-up reliability differs across patient subgroups. Sustainability should include monitoring for performance drift, stability across changing workflows, and the need for recalibration or controlled updating over time. These domains are intended to function as a minimal operational framework rather than a rigid universal checklist. Specific implementations may require additional task-specific measures, but a shared core set of process, safety, oversight, equity, and sustainability metrics can support more consistent comparison, evaluation, and reproducibility across different nephrology settings.

Two process measures are particularly central.

- Time-to-action quantifies the latency between recognition of a clinically meaningful signal and initiation of an appropriate response. Examples include time from AKI trajectory deviation to nephrology consultation, time from accelerated CKD decline to medication reassessment, or time from abnormal transplant surveillance results to clinician review. Time-to-action captures whether an agent meaningfully improves the tempo of care rather than merely generating information [45,46].
- For example, in AKI, time-to-action may be defined as the interval between algorithmic recognition of a concerning creatinine trajectory or urine output pattern and completion of a clinically appropriate response, such as nephrology consultation, medication review, fluid adjustment, or repeat laboratory testing. In this setting, measurement may be reported in minutes or hours, depending on the care environment, and can be compared across implementation periods to assess whether workflow-integrated systems reduce delays in clinical response. Similarly, in CKD, process-level endpoints may include time from identification of high-risk disease progression to nephrology referral, medication optimization, or repeat albuminuria and kidney function testing. In transplant care, comparable endpoints may include time from abnormal surveillance findings to clinician review or follow-up intervention. These examples illustrate how process measures can provide practical evidence of whether clinical AI agents improve the timeliness and coordination of care beyond prediction alone.
- Clinician override patterns provide a pragmatic and interpretable measure of alignment, trust calibration, and safety [45,46]. Overrides should be analyzed contextually rather than treated as binary failures. High override rates may indicate poor prioritization or excessive autonomy, whereas persistently low override rates may reflect overly conservative behavior with limited clinical impact. Patterns of override across scenarios, clinician roles, and time offer actionable insight into whether agents are functioning as intended collaborators.

This set of metrics assesses whether an agent effectively converts recognition into action while preserving appropriate human control (Table 2).

### 8.3. Outcome Metrics as Confirmatory Evidence

Clinical outcomes remain essential, but their role in agent evaluation should be confirmatory rather than primary. Outcomes such as hospitalization rates, dialysis complications, graft dysfunction, or eGFR decline slopes are influenced by multiple factors beyond the agent itself and often require prolonged observation periods [4,105].

When used appropriately, outcome metrics validate that improvements in process translate into meaningful patient benefit. However, failure to demonstrate short-term outcome differences should not be interpreted as agent failure if process-level improvements are evident [106]. Expecting immediate outcome separation risks penalizing systems designed to improve reliability and timeliness rather than override clinical judgment.

### 8.4. Safety Evaluation Beyond Adverse Events

Safety assessment for clinical AI agents must extend beyond traditional adverse event reporting [97,107]. Because agents influence care trajectories over time, evaluation should include near-miss detection, failure-to-escalate events, inappropriate persistence of outdated recommendations, and breakdowns in human–machine handoff [108].

Particular attention should be paid to how agents behave under uncertainty. Safe agents should modulate autonomy when confidence is low, escalate appropriately, and defer to human judgment rather than persist with brittle recommendations [109,110]. These behaviors are not captured by conventional performance metrics and must be evaluated explicitly.

### 8.5. Trust, Equity, and Sustainability

Clinician trust is both a determinant and an outcome of effective agent deployment. In addition to override frequency, qualitative assessment of clinician experience should inform evaluation frameworks, capturing usability, cognitive burden, and perceived reliability [111].

Equity evaluation is essential. Because agents shape workflows rather than isolated decisions, inequities may manifest through differential escalation timing, access to specialty care, or follow-up reliability across populations [112,113]. These effects will not be detected through aggregate discrimination metrics and require deliberate analysis.

Equity considerations are an important component of clinical AI agent deployment. Healthcare data often reflect underlying structural differences in access to care, diagnostic testing, documentation practices, and social determinants of health [112,113]. As a result, datasets used to develop and operate clinical AI agents may contain systematic gaps that mirror existing disparities in healthcare delivery. If not addressed, agent-based systems could unintentionally reproduce or amplify inequities in clinical attention, escalation, or follow-up [112,113].

These risks are particularly relevant for workflow-integrated systems that continuously interpret patient data and prioritize actions. Patients with fragmented care histories, limited laboratory monitoring, or incomplete documentation may appear artificially stable to automated systems, potentially delaying escalation or clinical review [112,113]. Conversely, patients who undergo more frequent testing may receive disproportionate system attention.

To mitigate these risks, clinical AI agents should be developed within equity-aware governance frameworks that assess training data representativeness and monitor performance across patient populations [112,113]. Evaluation should include analysis of escalation patterns, alerts, referrals, and time-to-action metrics across demographic and clinical subgroups to identify unintended disparities.

Equity monitoring should also continue after deployment. Because agentic systems interact dynamically with evolving clinical workflows and data streams, ongoing auditing and governance oversight are necessary to ensure that workflow-integrated AI improves care reliability without reinforcing existing healthcare inequities [112,113].

Finally, sustainability must be addressed. Clinical AI agents operate in dynamic environments where guidelines evolve, workflows change, and patient populations shift [114]. Ongoing monitoring for performance drift, controlled updating, and governance-guided adaptation should be considered integral components of evaluation rather than post-deployment considerations [46,110].

## 9. Implications for Future Research

From a clinical perspective, the emergence of clinical AI agents has the potential to influence several aspects of kidney care delivery [19]. By integrating longitudinal patient data with clinical knowledge and workflow-aware reasoning, these systems may support earlier identification of clinical deterioration, improved coordination of multidisciplinary care, and more consistent implementation of guideline-based interventions. In chronic kidney disease management, for example, agent-based systems could assist clinicians in tracking disease trajectories, identifying opportunities for therapeutic optimization, and ensuring timely monitoring of laboratory parameters and medications. Similar approaches may support dialysis management, transplantation follow-up, and the monitoring of complex immune-mediated kidney diseases, where care often requires coordination among multiple specialists and healthcare settings.

However, translating these concepts into clinical practice will require careful attention to governance, safety, and workflow integration. Clinical AI agents should be designed to support, rather than replace, clinician decision-making, operating within clearly defined safety constraints and human oversight. Integration with electronic health record systems, transparent decision logging, and mechanisms for clinician feedback and override will be essential to ensure safe implementation and to maintain trust in these technologies.

Furthermore, the implementation of clinical AI agents carries organizational implications that extend beyond algorithmic performance. Successful deployment requires not only technical infrastructure but also workflow redesign, institutional governance, and careful consideration of implementation costs [19]. In practice, early adoption will likely focus on areas where agents can reduce administrative workload, support longitudinal monitoring, and improve coordination of routine care tasks rather than adding new cognitive demands for clinicians. Examples include automated monitoring of laboratory trends, identification of missed follow-up tests, preparation of draft clinical documentation, and coordination of routine referrals or surveillance tasks.

The introduction of agentic systems may also influence professional roles within nephrology teams [18]. Rather than replacing clinicians, clinical AI agents are expected to function as workflow-support tools that help redistribute routine information management tasks while preserving clinician authority over diagnostic and therapeutic decisions. This redistribution may allow clinicians to focus more on complex clinical reasoning, patient communication, and multidisciplinary coordination. At the organizational level, successful integration will require training, governance oversight, and iterative evaluation to ensure that these systems reduce rather than exacerbate cognitive burden in already saturated clinical environments.

Furthermore, the concept of clinical AI agents should be understood not as a purely theoretical construct but as an implementation-oriented framework that defines how AI systems may be designed to participate safely in clinical care processes [4,19]. In this context, the goal is not to propose autonomous decision-making systems but to outline the architectural, governance, and evaluation principles required for workflow-integrated intelligence in healthcare. By framing AI as a structured participant in care delivery, the clinical AI agent paradigm provides a foundation for designing systems that support longitudinal monitoring, task coordination, and guideline-consistent clinical workflows.

Importantly, operational implementation of clinical AI agents will likely occur through staged deployment [4,19]. Early systems are expected to focus on constrained workflow-support functions such as monitoring longitudinal patient data, identifying missed follow-up actions, organizing clinical information, and preparing documentation for clinician review. These roles allow agent-based systems to reduce administrative burden and improve care coordination while maintaining clear human oversight of clinical decisions.

As technical infrastructure, interoperability standards, and governance frameworks mature, these systems may progressively support more complex workflow coordination across multidisciplinary teams [4,19]. The conceptual framework described in this review therefore serves as a practical roadmap for future system development, providing guidance on architecture, safety boundaries, evaluation metrics, and organizational integration required for responsible deployment of clinical AI agents in nephrology.

Several limitations of this review should also be acknowledged. First, the manuscript represents a conceptual synthesis of the emerging literature rather than a formal systematic review or meta-analysis, and therefore the included references were selected to illustrate key themes rather than to provide an exhaustive summary of all available studies. Second, the field of agentic AI in healthcare is rapidly evolving, and many proposed architectures and applications remain at an early stage of development, with limited pilot implementation or prospective trial data currently available [4,19]. As a result, some aspects of the framework described in this review remain conceptual and will require future empirical validation. Finally, the clinical impact of such systems will depend not only on technical performance but also on successful integration into clinical workflows, regulatory oversight, and acceptance by clinicians and patients.

These considerations require a reframing of how agentic systems are studied. Predictive accuracy should be treated as necessary but insufficient [4,8]. Process reliability, timeliness of action, safety under uncertainty, and trust calibration must move to the center of evaluation. Future work should include pilot implementations, prospective studies, and controlled trials to better distinguish conceptual promise from demonstrated real-world feasibility in nephrology practice.

Clinical AI agents should be judged not by how well they predict outcomes, but by how effectively they change care processes in ways that improve patient-centered outcomes while preserving clinician accountability [4,8].

## 10. The Importance of Staged Deployment

Successful integration of clinical AI agents requires staged deployment, beginning with advisory and coordination roles before progressing to limited, well-defined autonomous actions [115,116]. Early implementations should emphasize transparency, auditability, and human-in-the-loop control, allowing institutions to calibrate trust, refine escalation thresholds, and identify failure modes. Over time, as evidence accumulates and governance matures, agent responsibilities can expand in a controlled manner [115,116]. This incremental approach aligns with both clinical culture and regulatory expectations, reducing risk while enabling innovation.

These considerations reinforce the central thesis of this review: the value of agentic AI in nephrology lies not in technical sophistication alone but in its alignment with real-world workflows, clinical accountability, and longitudinal care delivery.

## 11. Conclusions

Clinical AI agents represent an important conceptual evolution in the application of AI to nephrology. Moving beyond prediction-based models, agentic systems offer the potential to integrate longitudinal patient data, clinical knowledge, and workflow-aware reasoning to support coordinated clinical actions across the kidney care continuum. When designed with appropriate safety constraints, governance frameworks, and human oversight, these systems may help translate data-driven insights into more reliable, timely, and patient-centered care.

Future work should focus on developing and evaluating clinical AI agents as workflow-integrated socio-technical systems rather than isolated predictive algorithms. Research priorities include assessing their impact on clinical workflows, time-to-action, clinician trust, safety, and patient outcomes, while ensuring transparency, accountability, and equitable implementation. Continued interdisciplinary collaboration between clinicians, health informatics researchers, regulators, and healthcare systems will be essential to guide the responsible development and clinical integration of these emerging technologies in nephrology.

## Figures and Tables

**Table 1:** Distinguishing Artificial Intelligence Paradigms in Nephrology.

Feature	Predictive ML	CDSS	LLM Chatbot	Clinical AI Agent
Primary function	Risk prediction or classification at discrete time points	Rule-based alerts and guideline reminders	Conversational information retrieval and text generation	Workflow-integrated clinical reasoning and task coordination
Temporal continuity	Absent	Absent	Limited	Present
Autonomous actions	Absent	Absent	Absent	Limited and constrained by human oversight, institutional policy, and regulatory requirements *
Workflow integration	Absent	Limited	Absent	Present
Goal-directed behavior	Absent	Absent	Absent	Present
Learning from outcomes	Limited	Absent	Absent	Present
Interaction with clinicians	Passive output requiring clinician interpretation	Alert-based interaction triggered by predefined rules	Prompt-based conversational interaction	Bidirectional interaction embedded within clinical workflows

* In this context, autonomy refers to the ability of clinical AI agents to perform predefined operational tasks within established safety boundaries, such as monitoring clinical data streams, generating alerts, organizing longitudinal clinical information, or initiating workflow-support actions (e.g., preparing documentation, suggesting follow-up testing, or prompting care coordination). Clinical decisions that directly influence diagnosis, treatment selection, or patient management require clinician review, confirmation, and accountability.

**Table 2:** Evaluating Clinical AI Agents in Nephrology.

Domain	Representative Metrics
Process performance	Time-to-action; response latency; completion of intended workflow steps; proportion of actionable outputs leading to clinical response
Safety	Near-miss detection; failure-to-escalate events; inappropriate recommendations or actions; breakdowns in human–machine handoff
Human oversight	Frequency and context of clinician overrides; trust calibration; clinician acceptance of agent-supported actions
Equity	Differential performance, escalation timing, and follow-up reliability across patient populations
Sustainability	Drift detection; performance stability over time; recalibration needs; adaptive performance monitoring

## Data Availability

No new data were created or analyzed in this study. Data sharing is not applicable to this article.

## Abbreviations

The following abbreviations are used in this manuscript:

AI	Artificial intelligence
AKI	Acute kidney injury
AUROC	Area under the receiver operating characteristic curve
CDSS	Clinical decision support system
CKD	Chronic kidney disease
CRRT	Continuous renal replacement therapy
EHR	Electronic health record
FHIR	Fast healthcare interoperability resources
LLM	Large language model
ML	Machine learning
RAG	Retrieval-augmented generation
SaMD	Software as a medical device
